# *Giardia duodenalis *assemblages and *Entamoeba *species infecting non-human primates in an Italian zoological garden: zoonotic potential and management traits

**DOI:** 10.1186/1756-3305-4-199

**Published:** 2011-10-12

**Authors:** Federica Berrilli, Cristina Prisco, Klaus G Friedrich, Pilar Di Cerbo, David Di Cave, Claudio De Liberato

**Affiliations:** 1 Dipartimento di Sanità Pubblica e Biologia Cellulare, Università di Roma Tor Vergata, Rome, Italy; 2 Fondazione Bioparco, Viale del Giardino Zoologico, 100197 Rome, Italy; 3 Istituto Zooprofilattico Sperimentale delle Regioni Lazio e Toscana, Rome, Italy

## Abstract

**Background:**

*Giardia duodenalis *and *Entamoeba *spp. are among the most common intestinal human protozoan parasites worldwide and they are frequently reported in captive non-human primates (NHP). From a public health point of view, infected animals in zoos constitute a risk for animal caretakers and visitors. In this study we carried out the molecular identification of *G. duodenalis *and *Entamoeba *spp. from nine species of primates housed in the zoological garden of Rome, to better ascertain their occurrence and zoonotic potential.

**Results:**

*G. duodenalis *was found only in *Lemur catta *(47.0%). *Entamoeba *spp. were detected in all species studied, with the exception of *Eulemur macaco *and *Varecia rubra*. The number of positive pools ranged from 5.9% in *L. catta *to 81.2% in *Mandrillus sphinx; *in *Pan troglodytes *the observed prevalence was 53.6%. A mixed *Entamoeba*-*Giardia *infection was recorded only in one sample of *L. catta*. All *G. duodenalis *isolates belonged to the zoonotic assemblage B, sub assemblage BIV. Three *Entamoeba *species were identified: *E. hartmanni*, *E. coli *and *E. dispar*.

**Conclusions:**

Our results highlight the importance of regularly testing animals kept in zoos for the diagnosis of zoonotic parasites, in order to evaluate their pathogenic role in the housed animals and the zoonotic risk linked to their presence. A quick detection of the arrival of pathogens into the enclosures could also be a prerequisite to limit their spread into the structure via the introduction of specific control strategies. The need for molecular identification of some parasite species/genotype in order to better define the zoonotic risk is also highlighted.

## Background

Protozoa are the most common parasites in captive non-human primates (NHP). Amongst others, *Giardia duodenalis *and *Entamoeba *spp. are frequently reported [1-3]; the simplicity of their monoxenous life cycle, the low infective dose and the short prepatent period facilitate their dispersal among captive animals once they have entered the enclosures (cages, pens, etc.) [4,5].

The relevance of *Giardia *and *Entamoeba *infections in zoo animals goes beyond their clinical effects. From a public health point of view, these protozoa have high zoonotic potential, being among the most common intestinal human parasites worldwide [6-8]. Infected NHP in zoos constitute a risk for animal caretakers [9,10] and possibly people visiting the zoological gardens. On the other hand, infected people could be the source of infection for the captive NHP via water and/or food contamination. Hence, the epidemiological relevance of gaining a better understanding of the transmission patterns of these pathogens in and from zoo facilities.

From a management point of view, new threats have arisen in the last few years due to the tendency to reproduce habitats as similar as possible to the natural ones. In particular, the removal of the cement as flooring and the use of distributing food mixed in the litter to elicit natural seeking behaviours, has facilitated faecal contamination and made disinfection of cages and pens very difficult, resulting in the creation of habitats suitable for parasite survival and transmission.

*G. duodenalis *is known to provoke gastrointestinal disorders in NHP [10,11]. This species includes at least seven genotypes or assemblages (A→G), assemblages A and B being detected both in humans and NHP [12,13]. Regarding the genus *Entamoeba*, in a recent study [14] six species (*E. histolytica, E. dispar*, *E. moshkovskii*, *E. hartmanni*, *E. coli *and *E. polecki*-like organisms) were recorded in captive NHP in Belgium and The Netherlands. The clinical relevance of *Entamoeba *spp. is more difficult to ascertain in NHP. Several species are considered non-pathogenic, while *E. histolytica *and its virulent variant *E. nuttalli *are known to provoke severe and sometimes lethal intestinal and extra-intestinal disorders in monkeys and apes [2,15-17]. Problems arise in *E. histolytica *diagnosis, because of the impossibility via conventional microscopy to distinguish between this species and the non-pathogenic *E. dispar *and *E. moshkovskii*. Hence, in the presence of a copro-parasitological diagnosis of amoebiasis, there is justification for using PCR to rule out infection with the pathogenic and potentially lethal species [18-21].

In the literature, there is no data regarding intestinal protozoa occurrence and molecular identification in NHP from Italian zoos. Following detection of *G. duodenalis *and *Entamoeba *spp. during routine copro-parasitological examinations, a study was carried out aimed at the molecular identification of *G. duodenalis *assemblages and *Entamoeba *species in NHP housed in the zoological gardens of Rome, to better define their presence and to understand their zoonotic potential.

## Methods

### Study site and Sampling

The Bioparco is one of the oldest zoological gardens in Europe, founded in 1911. It is located in the city centre of Rome (Italy), covering an area of 18 ha and housing about 1000 specimens belonging to almost 200 species comprising mammals, birds and reptiles. It was designed in accordance with the new concept of "Zoo without bars" to improve animal welfare. Animals live in large spaces with reconstruction of the natural habitats suitable for each species. To avoid contact with people, glass screens or ditches border animal cages and pens.

Nine species and 3 families of NHP were involved in this study, the families being: *Lemuridae *(prosimians), *Cercopithecidae *(Old Word monkeys) and *Hominidae *(apes) (Table 1). At the Bioparco, monkeys and apes are kept in monospecific groups of 2-98 individuals sharing the same pens. All NHP are housed in cages and pens littered with natural materials such as ground bark and they are fed mixing the food with the litter.

**Table 1:** Number of NHP specimens per cage; number of pools of faeces tested microscopically and number of pooled positive samples for *Entamoeba *spp. and *Giardia duodenalis*.

*Scientific name*	Common name	N° of specimens/cage	N° pool of faeces	*Entamoeba *sp. N°positive (%)	G. duodenalis *N°positive (%)*
Lemuridae					

*Lemur catta*	Ring-tailed lemur	10	17	1 (5.9)	8 (47.0)

*Eulemur macaco*	Black lemur	3	4	0	0

*Varecia rubra*	Red ruffed lemur	3	5	0	0

Cercopithecidae					

*Cercocebus torquatus*	Collared mangabey	5	10	6 (60.0)	0

*Chlorocebus aethiops*	Vervet monkeys	2	5	4 (80.0)	0

*Macaca fuscata*	Japanese macaque	98	22	12 (54.5)	0

*Mandrillus sphinx*	Mandrill	18	16	13 (81.2)	0

Hominidae					

*Pan troglodytes*	Common chimpanzee	5	41*	22 (53.6**)	0

*Pongo pygmaeus*	Bornean orangutan	3	13	6 (46.1)	0

Total			133	64 (48.1)	8 (6.0)

* Individual faecal samples

** Prevalence

Due to the group housing, copro-parasitological analysis was performed on pools of faeces collected from the litter, with the exception of *P. troglodytes*, where individual sampling was possible.

### Coprological examination

All the samples were sent to the laboratory of Parasitology of the IZSLT for routine copro-parasitological diagnosis. Faecal samples were examined for parasitic protozoa cysts and/or trophozoites using the wet mount Lugol's iodine staining method and the formol ethyl-acetate concentration technique [22,23]. In case of ambiguous results obtained using microscopy, *G. duodenalis *infection was confirmed via a commercial immunofluorescence kit, (MERIFLUOR^® ^*Cryptosporidium/Giardia*, Meridian Bioscience, Inc.).

Samples positive for *G. duodenalis *or *Entamoeba *spp. cysts were transferred to the University of Rome Tor Vergata for molecular characterization.

### Molecular analysis and sequencing

Genomic DNA was extracted from faecal samples using the QIAamp DNA Stool Mini Kit (Qiagen, Italy) following manufacturer's instructions. Molecular analysis was carried out by amplifying an 18S rRNA genus fragment both for *G. duodenalis *and *Entamoeba *spp.

For *Giardia*, a nested PCR procedure was performed to amplify a 130 bp region using the primers RH4 and RH11 [24] for the first step and the primers GiarR and GiarF in the second amplification round [25].

The amplification of the specific fragment of *Entamoeba *spp. was obtained using the primers JVC and DSPR2 [26], which are also able to detect the virulent variant of *E. histolytica*, *E. nuttalli*, observed in NHP [17]. The amplicons were from 622 to 667 bp long depending on the species.

To confirm *Giardia *18S rRNA genetic identity of the samples, an additional analysis was performed by sequencing the triose phosphate isomerase (*tpi) *and glutamate dehydrogenase (*gdh*) loci. To amplify the *tpi *fragment, a nested PCR procedure was used to obtain a 530 bp region using the primers AL3543 and AL3546 for the first step and AL3544 and AL3545 for the second one [27]. For *gdh*, a semi-nested PCR was carried out to amplify a 432 bp fragment with the primers GDHeF and GDHiR in the primary reaction, and GDHiF and GDHiR in the secondary [28]. In all PCR reactions, positive and negative controls were added.

Amplicons were visualized by electrophoresis on SYBR Safe DNA-stained 1% agarose gel (Invitrogen). Bands of the predicted sizes were excised and DNA was purified with the NucleoSpin Extract II purification kit (Macherey-Nagel, GmbH & Co. KG, Germany) and sequenced in both directions by Bio-Fab Research (Italy).

Sequences were edited with FinchTV 1.4 Software (Geospiza, Inc, Seattle, WA) by the analysis of chromatograms. Consensus sequences were obtained using ClustalW2 Multiple Sequence Alignments and queried against known sequences of GenBank database using BLAST. Molecular characterization of *Entamoeba *spp. and *G. duodenalis *isolates was based on both sequence comparison and phenetic analysis. Phenetic analyses were conducted with the MEGA (version 4.0) software. A distance-based analysis was carried out using the maximum composite likelihood method, and trees were constructed by the Neighbour Joining (NJ) algorithm. Bootstrap values were calculated by analysing 500 replicates.

## Results

Between January 2010 and March 2011, 133 faecal samples were analysed microscopically for parasites (Table 1). *G. duodenalis *was found only in *L. catta*, in 47% of tested pooled samples. *Entamoeba *spp. were detected in all the considered species, except for *E. macaco *and *V. rubra*; number of positive pooled samples ranged from 5.9% in *L. catta *to 81.2% in *Mandrillus sphinx*. In *P. troglodytes *the observed prevalence (individual sampling) was 53.6%.

A mixed *Entamoeba*-*Giardia *infection was recorded only in one sample of *L. catta*. All animals showed no symptoms.

### Molecular analysis

#### Amplification of the 18S-rRNA, gdh and tpi *G. duodenalis *genes

Of the eight *Giardia *positive isolates from *L. catta *tested at the *18S-rRNA*, *gdh *and *tpi *loci, all three genes were successfully amplified and sequenced from 4 isolates, while one or two genes were amplified from the other four. The combined results of the multilocus analyses are summarized in Table 2.

**Table 2:** Summary of multilocus genotyping results of *Giardia duodenalis *samples from *Lemur catta *at the level of assemblage and sub-assemblage.

Isolate code	*18S-rRNA*	*gdh*	*tpi*
L1	B	B(IV)	B(IV)

L2	B	B(IV)	B(IV)

L8A	B	B(IV)	B(IV)

L8B	B	B(IV)	B(IV)

L8C	B	-	B(IV)

L8D	B	-	-

L10A	B	-	-

L10B	B	-	-

**Figure 1:**
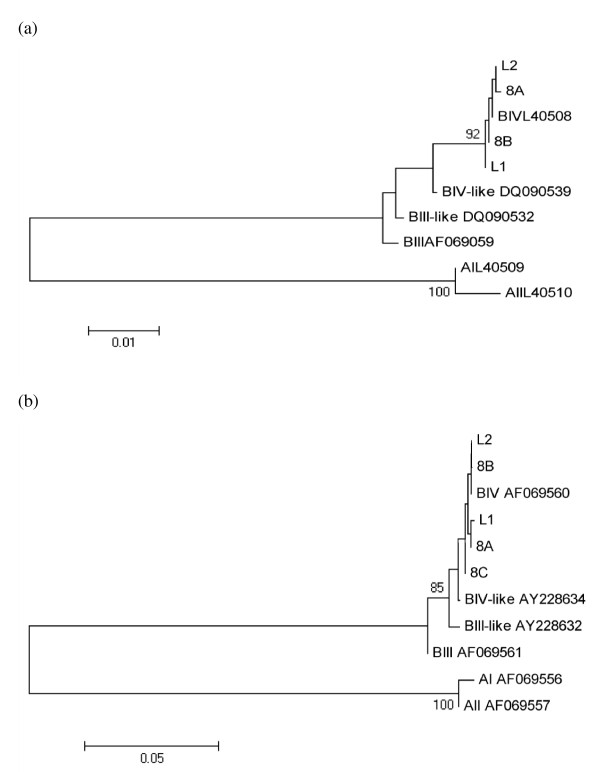
**Phenetic relationships of *G. duodenalis *inferred by NJ analysis of the *gdh *(a) and *tpi *(b) loci**. Only bootstrap values > 70 are indicated. The Accession Numbers utilized for *gdh *were L40509 (AI), L40510 (AII), AF069059 (BIII), DQ090539 (BIII-like), L40508 (BIV), DQ090532 (BIV-like) and those ones for *tpi *were AF069556 (AI), AF069557 (AII), AF069561 (BIII), AY228632 (BIII-like), AF069560 (BIV), AY228634 (BIV-like).

Sequences obtained were identified at the *18S-rRNA *locus as assemblage B, by comparing to GenBank sequences of *Giardia *genotypes (Accession Numbers: AF199446 (assemblage A), AF199447 (assemblage B), AY775200 (assemblage C), AY775199 (assemblage D), AY297957 (assemblage E), AF199444 (assemblage F) and AF199450 (assemblage G). The bootstrap consensus trees for both *gdh *and *tpi *genes obtained by NJ method yielded one monophyletic group corresponding to the assemblage B, sub assemblage BIV which included *Giardia *sequences from *L. catta *(Figure 1). Mixed assemblages, showing double peaks at the diagnostic positions at the three loci in the chromatograms, were not observed. Consensus trees obtained by the phenetic analysis confirmed this result.

#### Amplification of the 18S-rRNA *Entamoeba *genes

**Figure 2:**
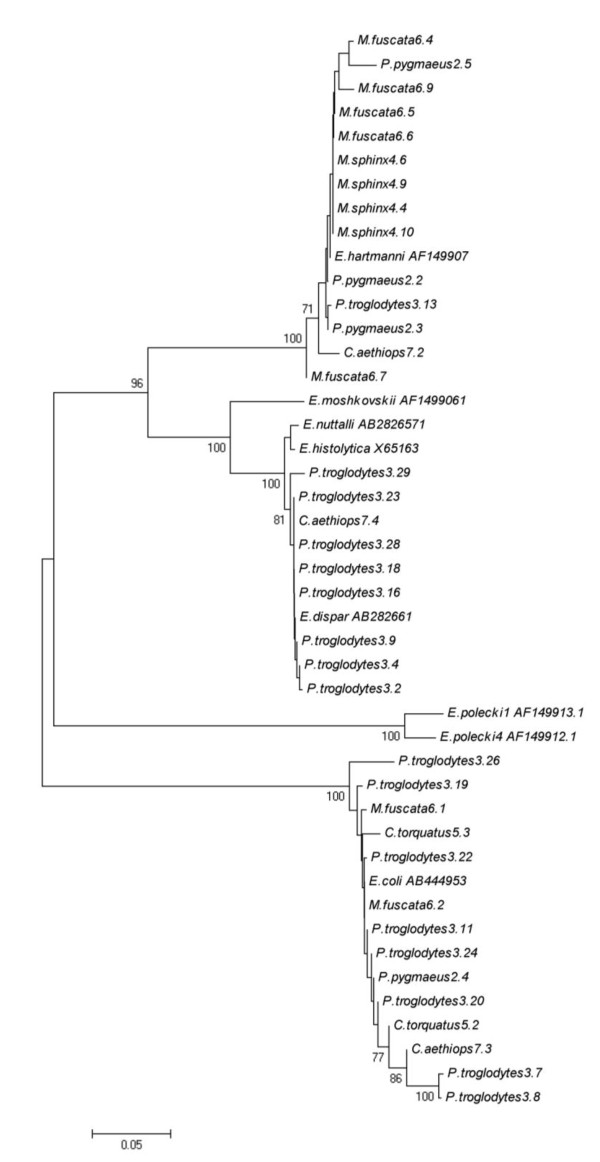
**Phenetic relationships of *Entamoeba *spp. inferred by NJ analysis of the 18S rRNA genus**. Only bootstrap values > 70 are indicated. The Accession Numbers utilized for the identification of *Entamoeba *spp. were AB282657 (*E. nuttalli*), X65163 (*E. histolytica*), AB282661 (*E. dispar*), AB444953 (*E. coli*), AF149907 (*E. hartmanni*), AF149906 (*E. moshkoskii*), AF149913 (*E. polecki-like *variant 1) and AF149912 (*E. polecki-like *variant 4).

The results revealed the presence of *Entamoeba *DNA in 45 out of 64 samples. In total three *Entamoeba *species were identified: *E. hartmanni *(31.1%), *E. coli *(31.1%) and *E. dispar *(20.0%) (Table 3). The assignment of the *Entamoeba *species within the hosts is reported in the constructed phenetic tree (Figure 2). No mixed infections were detected. Non-interpretable sequences were obtained from 8 of the amplified samples, but showed homology to *Entamoeba *spp. For 4 samples, DNA amplification was unsuccessful; for 15 sequences homology was found to DNA sequences from plants, ascomycetes and zygomycetes (*Malus domestica*, *Pyrus communis*, *Sordaria fimicola*, *Neurospora crassa*, *Helicostylum pulchrum*, *Sporodiniella umbellata*).

**Table 3:** Summary of molecular identification of *Entamoeba *species.

*Host species*	*E. hartmanni *N°positive (%*)	*E. dispar *N°positive (%*)	*E. coli *N°positive (%*)	*Entamoeba *sp. N°positive (%*)
*Cercocebus torquatus*	0	0	2 (66.7)	1 (33.3)

*Chlorocebus aethiops*	1 (33.3)	1 (33.3)	1 (33.3)	0

*Macaca fuscata*	5 (62.5)	0	2 (25.0)	1 (12.5)

*Mandrillus sphinx*	4 (80.0)	0	0	1 (20.0)

*Pan troglodytes*	1 (4.5)	8 (36.3)	8 (36.3)	5 (22.7)

*Pongo pygmaeus*	3 (75.0)	0	1 (25.0)	0

Total	14 (31.1)	9 (20.0)	14 (31.1)	8 (17.8)

* Calculated as follows: number of positive samples for the species/number of *Entamoeba *positive samples at molecular analysis

## Discussion

To the best of our knowledge, these are the first data regarding *G. duodenalis *and *Entamoeba *spp. in NHP housed in a zoological garden in Italy. Of particular concern are the levels of infection recorded (47% for *G. duodenalis *in *L. catta *and up to 81.2% for *Entamoeba *spp. in *M. sphinx*) and the report of zoonotic assemblages and species for both taxa. These findings appear comparable with previous data available in the literature. Regarding *Entamoeba *spp., Levecke et al. [2] reported values of prevalence among Old World monkeys ranging from 30% and 100% and among apes ranging from 0% and 100%, with higher values observed in *M. sphinx *and *P. pygmaeus*. Lower levels of infection, with overall values ranging from 29.3% for monoinfections to 51.9% for mixed infections, were recorded by Levecke et al. [14] in 36 species of NHP housed in nine zoos in Belgium and The Netherlands.

With respect to *G. duodenalis*, its report from captive *L. catta *confirms previous studies, where values of prevalence as high as 94% were recorded [2,13]. Interestingly, Villers et al. [29], in a comparative study on intestinal parasites of wild and captive *L. catta*, found this parasite only in the captive ones. Also, lack of symptoms in *L. catta *infected with *Giardia *is a recorded occurrence [29,30]. From a molecular point of view, the identification of assemblage B confirms the report of Beck et al. [30] in *L. catta *from Zagreb zoological garden and of previous data about this parasite from NHP [31-33]. Zoonotic assemblages A and B have also been described in NHP by Graczyk et al. [34] and Vitazkova and Wade [12].

Captive animals in zoos are exposed to various stresses and pathogens, resulting in an increased probability of becoming infected with parasites and of developing high parasite burdens [35]. Exposure to strict non-natural contact with specimens of the same as well as of other species, humans included, enhances their probability of becoming infected with zoonotic agents [30]. Moreover, questions arise about the route of entry of these pathogens in a theoretically isolated enclosure. Identification of transmission routes is always difficult [30], and only a hypothesis can be postulated: monoxenous protozoa following the faecal-oral transmission route like *G. duodenalis *and *Entamoeba *spp. could arrive in a zoo via contaminated vegetables fed to the animals, via water supplies, carried (even mechanically) by free ranging mammals and birds entering cages and pens and with the intake of new animals arriving from other zoos. In the present study, the presence of *Giardia *only in *L. catta *could be related to the recent (May 2007) admission of the colony of Ring-tailed lemur in the Bioparco, probably including infected but asymptomatic specimens. The hypothesis of a single and confined intake event is supported by the molecular data obtained by the multi-locus sequencing analysis, never recording mixed *Giardia *assemblage infections. On the basis of the different nature of the genes (multicopy/single copy), a different amplification rate at the three loci is frequently reported in the literature. However, despite regarding only 5 isolates of the 8 obtained, all of them were identified as assemblage B, sub-assemblage BIV, in contrast to other studies where isolates from NHP were identified as BIII, BIII-like and/or BIV-like at either the *gdh *or the *tpi *gene [32,36]. The lack of symptoms in the animals made the intake of the parasite easier, despite the quarantine regulations adopted in the zoo. More difficult to hypothesize is the route of entry of *Entamoeba *spp., as these parasites were widespread among many different host species of NHP and their intake could have occurred a long time ago, giving rise to their establishment in the zoological garden and subsequent spread to many susceptible host species.

Regarding molecular analysis of *Entamoeba *isolates, discrepancy between microscopy and genetic analysis results could be due to both inhibitory problems of PCR and to low sensitivity/specificity of microscopy [14]. As expected, since all animals were asymptomatic, *E. histolytica *and the virulent variant observed in NHP, *E. nuttalli*, were not detected. The other species, *E. hartmanni, E. dispar *and *E. coli*, are usually recovered and considered commensals.

Mixed infections are frequently reported in the literature. For *G. duodenalis*, Levecke et al. [13] showed mixed assemblages in 32.7% of NHP samples, including Ring-tailed lemurs. Also for *Entamoeba *spp., in a later study by Levecke et al., [14], most of NHP samples (51.9%) carried more than one species. The absence of mixed infections in our results could be related to the molecular approach used in the study, since more sensitive methods have been recently suggested [37,14]. However, concerning *G. duodenalis*, our data could likely be related to the low number of positive samples and/or to the arrival of this parasite with *L. catta *in a single intake event.

Regardless of their pathogenic potential for humans, the presence of *Entamoeba *and *Giardia *raises questions about the risk linked to zoo keeping operations in primate enclosures. In particular, all parasites found in the present study (*E. dispar*, *E. coli*, *E. hartmanni *and, especially, *G. duodenalis *assemblage B sub-assemblage BIV) warrant the maximum attention regarding the possibility of transmission among animals and their caretakers and can be considered potentially zoonotic pathogens. Zoo animals infected with zoonotic parasites also pose a problem concerning habitat contamination with cysts and eggs potentially able to infect people or other animals, as demonstrated by the detection of *Giardia *cysts in water bodies in and close to a zoological garden in Malaysia [35].

It appears very difficult to control such a parasite once it gets into the structure. New systems in the pens' flooring and feeding modalities make the washing and disinfection of pens and cages more difficult and facilitate the parasite life cycle, with continuous re-infections of the housed animals. In the present study, repeated treatments of *L. catta *specimens were ineffective in erradicating the infection from the colony, with animals testing positive even immediately after treatments. In general, the pharmacological control of protozoa appears arduous, and, as a matter of fact, their presence in captive NHP, in the absence of clinical symptoms, is often accepted and they are managed rather than controlled.

The presence of parasites with direct life cycles in zoos raises many management problems, linked to the difficulty of preventing cyst/eggs transport from one enclosure to the other. The absence of *Giardia *positive samples in the examined species other than *L. catta*, allows us to suppose that the parasite was actually confined to the Ring-tailed lemur colony and that the prophylaxis measures implemented until now by the zoo veterinarians (disinfection of animal-keepers shoes, use of disposable gloves and instruments, etc.) were effective in avoiding the spreading of the infection to other cages and pens.

Finally, this study suggests the need to improve copro-parasitological diagnosis with molecular analysis, aimed at distinguishing among pathogenic/non pathogenic and zoonotic/non zoonotic species and assemblages and to improve sensitivity of tests carried out on animals kept in quarantine before their intake into zoo facilities, thus preventing the arrival of pathogens in these particular kinds of confined habitats.

## Conclusions

Our results highlight the need for regularly testing of animals kept in zoo facilities for the diagnosis of zoonotic parasites, in order to point out their eventual arrival in the enclosures and evaluate their pathogenic role in the housed animals and the zoonotic risk linked to their arrival. A quick detection of the arrival of pathogens into the enclosures could also be a prerequisite to limit their spread in the structure via the implementation of specific control strategies and could permit the identification of intake routes, thus allowing the introduction of specific prophylactic measures. The need for molecular identification of some parasite species/genotype in order to better define the risk linked to their presence in the zoo animals is also highlighted.

## List of abbreviations used

NHP: Non-human primates; IZSLT: Istituto Zooprofilattico Sperimentale delle Regioni Lazio e Toscana.

## Competing interests

The authors declare that they have no competing interests.

## Authors' contributions

FB designed and carried out molecular analysis and wrote the paper. CP carried out molecular analysis and drafted the manuscript. KGF and PDC carried out sample collection, contributed and commented on the paper. DDC drafted and commented on the manuscript. CDL designed sampling collection, carried out parasitological examinations and wrote the paper. All authors approved the final version of the manuscript.
